# 1,1′-{[1,4-Phenyl­enebis(methyl­ene)]bis­(­oxy)bis­(3,1-phenyl­ene)}diethanone

**DOI:** 10.1107/S1600536811045041

**Published:** 2011-11-02

**Authors:** Nassir N. Al-Mohammed, Yatimah Alias, Zanariah Abdullah, Hamid Khaledi

**Affiliations:** a Department of Chemistry, University of Malaya, 50603 Kuala Lumpur, Malaysia

## Abstract

In the title compound, C_24_H_22_O_4_, the centroid of the central benzene ring lies on a special position of 2/*m* site symmetry, while the terminal aromatic rings are located on a mirror plane. The central and terminal benzene rings are perpendic­ular to each other. In the crystal, the mol­ecules are connected *via* C—H⋯O hydrogen bonds into a three-dimensional polymeric structure. The network is further consolidated by a C—H⋯π inter­action.

## Related literature

For the related structure of the *o*-acetyl isomer, see: Al-Mohammed *et al.* (2011 ▶).
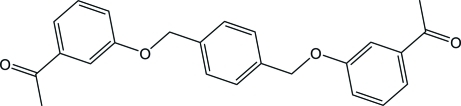


## Experimental

### 

#### Crystal data

C_24_H_22_O_4_
*M* *_r_* = 374.42
Monoclinic, 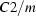

*a* = 20.6446 (9) Å
*b* = 7.0205 (4) Å
*c* = 6.5523 (3) Å
β = 93.083 (3)°
*V* = 948.29 (8) Å^3^
*Z* = 2
Mo *K*α radiation
μ = 0.09 mm^−1^
*T* = 100 K
0.27 × 0.15 × 0.05 mm

#### Data collection

Bruker APEXII CCD diffractometer
Absorption correction: multi-scan (*SADABS*; Sheldrick, 1996 ▶) *T*_min_ = 0.977, *T*_max_ = 0.996
3981 measured reflections
1120 independent reflections
872 reflections with *I* > 2σ(*I*)
*R*_int_ = 0.024

#### Refinement

*R*[*F* ^2^ > 2σ(*F* ^2^)] = 0.040
*wR*(*F* ^2^) = 0.107
*S* = 1.05
1120 reflections
83 parameters
H-atom parameters constrained
Δρ_max_ = 0.26 e Å^−3^
Δρ_min_ = −0.21 e Å^−3^

### 

Data collection: *APEX2* (Bruker, 2007 ▶); cell refinement: *SAINT* (Bruker, 2007 ▶); data reduction: *SAINT*; program(s) used to solve structure: *SHELXS97* (Sheldrick, 2008 ▶); program(s) used to refine structure: *SHELXL97* (Sheldrick, 2008 ▶); molecular graphics: *X-SEED* (Barbour, 2001 ▶); software used to prepare material for publication: *SHELXL97* and *publCIF* (Westrip, 2010 ▶).

## Supplementary Material

Crystal structure: contains datablock(s) I, global. DOI: 10.1107/S1600536811045041/is2798sup1.cif

Structure factors: contains datablock(s) I. DOI: 10.1107/S1600536811045041/is2798Isup2.hkl

Additional supplementary materials:  crystallographic information; 3D view; checkCIF report

## Figures and Tables

**Table 1:** Hydrogen-bond geometry (Å, °) *Cg* is the centroid of the central benzene ring.

*D*—H⋯*A*	*D*—H	H⋯*A*	*D*⋯*A*	*D*—H⋯*A*
C5—H5⋯O2^i^	0.95	2.50	3.435 (2)	170
C11—H11⋯O1^ii^	0.95	2.54	3.4215 (16)	155
C6—H6⋯*Cg*^i^	0.95	2.95	3.8469 (19)	157

Symmetry codes: (i) 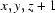; (ii) 
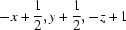
.

**Fig. 1.**
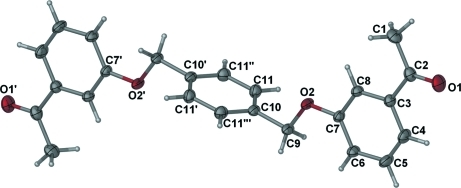
Molecular structure of the title compound with displacement ellipsoids drawn at 50% probability level. Hydrogen atoms are drawn as spheres of arbitrary radius. [Symmetry codes: ' = -x+1, -y, -z; " = -x + 1, y, -z; "' = x, -y, z.]

## supplementary crystallographic information

### Comment

We have recently reported the crystal structure of the *o*-acetyl isomer of the title compound (Al-Mohammed *et al.*, 2011). In the present molecule all the non-H atoms, except for C11, lie on a mirror plane and the centroid of the central benzene ring is placed on a special position of 2/m site symmetry. In the crystal, the molecules are linked through C—H···O bonds (Table 1) into a three-dimensional polymeric structure. The network is further consolidated by C—H···π interactions [C6 ···*Cg* = 3.8469 (19) Å where *Cg* is the centroid of the central benzene ring]

### Experimental

To a mixture of α,α'-dibromo-*p*-xylene (1 g, 3.8 mmol) and potassium carbonate (1.05 g, 7.57 mmol) in dry acetone (25 ml), 3'-hydroxyacetophenone (1.03 g, 7.57 mmole) was added. The mixture was refluxed for 2 days. The solvent was then evaporated under reduced pressure and the crude material was extracted by dichloromethane (3 × 25 ml). The combined organic layers was washed with water and brine and dried over sodium sulfate. The solvent was then evaporated the solid was re-crystallized from chloroform to give off-white crystals of the title compound.

### Refinement

Hydrogen atoms were placed at calculated positions and refined as riding atoms with C—H distances of 0.95 (aryl), 0.98 (methyl) and 0.99 (methylene) Å, and *U*_iso_(H) set to 1.2 (1.5 for methyl) *U*_eq_(carrier atoms). A rotating group model was used for the methyl group.

### Crystal data

**Table:**

C_24_H_22_O_4_	*F*(000) = 396
*M**_r_* = 374.42	*D*_x_ = 1.311 Mg m^−^^3^
Monoclinic, *C*2/*m*	Mo *K*α radiation, λ = 0.71073 Å
Hall symbol: -C 2y	Cell parameters from 1083 reflections
*a* = 20.6446 (9) Å	θ = 3.1–29.5°
*b* = 7.0205 (4) Å	µ = 0.09 mm^−^^1^
*c* = 6.5523 (3) Å	*T* = 100 K
β = 93.083 (3)°	Plate, colorless
*V* = 948.29 (8) Å^3^	0.27 × 0.15 × 0.05 mm
*Z* = 2	

### Data collection

**Table:**

Bruker APEXII CCD diffractometer	1120 independent reflections
Radiation source: fine-focus sealed tube	872 reflections with *I* > 2σ(*I*)
graphite	*R*_int_ = 0.024
φ and ω scans	θ_max_ = 27.0°, θ_min_ = 3.1°
Absorption correction: multi-scan (*SADABS*; Sheldrick, 1996)	*h* = −26→25
*T*_min_ = 0.977, *T*_max_ = 0.996	*k* = −8→8
3981 measured reflections	*l* = −8→8

### Refinement

**Table:**

Refinement on *F*^2^	Primary atom site location: structure-invariant direct methods
Least-squares matrix: full	Secondary atom site location: difference Fourier map
*R*[*F*^2^ > 2σ(*F*^2^)] = 0.040	Hydrogen site location: inferred from neighbouring sites
*wR*(*F*^2^) = 0.107	H-atom parameters constrained
*S* = 1.05	*w* = 1/[σ^2^(*F*_o_^2^) + (0.045*P*)^2^ + 0.5308*P*] where *P* = (*F*_o_^2^ + 2*F*_c_^2^)/3
1120 reflections	(Δ/σ)_max_ < 0.001
83 parameters	Δρ_max_ = 0.26 e Å^−^^3^
0 restraints	Δρ_min_ = −0.21 e Å^−^^3^

### Special details

**Table:**

Geometry. All e.s.d.'s (except the e.s.d. in the dihedral angle between two l.s. planes) are estimated using the full covariance matrix. The cell e.s.d.'s are taken into account individually in the estimation of e.s.d.'s in distances, angles and torsion angles; correlations between e.s.d.'s in cell parameters are only used when they are defined by crystal symmetry. An approximate (isotropic) treatment of cell e.s.d.'s is used for estimating e.s.d.'s involving l.s. planes.
Refinement. Refinement of *F*^2^ against ALL reflections. The weighted *R*-factor *wR* and goodness of fit *S* are based on *F*^2^, conventional *R*-factors *R* are based on *F*, with *F* set to zero for negative *F*^2^. The threshold expression of *F*^2^ > σ(*F*^2^) is used only for calculating *R*-factors(gt) *etc*. and is not relevant to the choice of reflections for refinement. *R*-factors based on *F*^2^ are statistically about twice as large as those based on *F*, and *R*- factors based on ALL data will be even larger.

### Fractional atomic coordinates and isotropic or equivalent isotropic displacement parameters (Å^2^)

**Table:**

	*x*	*y*	*z*	*U*_iso_*/*U*_eq_	Occ. (<1)
O1	0.09646 (6)	0.0000	0.6315 (2)	0.0420 (4)	
O2	0.35997 (5)	0.0000	0.31330 (17)	0.0303 (4)	
C1	0.11430 (9)	0.0000	0.2772 (3)	0.0379 (5)	
H1A	0.1278	−0.1190	0.2140	0.057*	0.50
H1B	0.1340	0.1082	0.2090	0.057*	0.50
H1C	0.0669	0.0109	0.2635	0.057*	0.50
C2	0.13609 (9)	0.0000	0.5003 (3)	0.0305 (4)	
C3	0.20700 (8)	0.0000	0.5583 (3)	0.0266 (4)	
C4	0.22729 (9)	0.0000	0.7659 (3)	0.0366 (5)	
H4	0.1961	0.0000	0.8674	0.044*	
C5	0.29237 (9)	0.0000	0.8226 (3)	0.0383 (5)	
H5	0.3058	0.0000	0.9636	0.046*	
C6	0.33890 (8)	0.0000	0.6764 (3)	0.0294 (4)	
H6	0.3838	0.0000	0.7170	0.035*	
C7	0.31899 (8)	0.0000	0.4707 (2)	0.0255 (4)	
C8	0.25302 (8)	0.0000	0.4124 (3)	0.0258 (4)	
H8	0.2396	0.0000	0.2715	0.031*	
C9	0.42832 (8)	0.0000	0.3689 (3)	0.0276 (4)	
H9A	0.4399	−0.1144	0.4514	0.033*	
C10	0.46469 (8)	0.0000	0.1763 (3)	0.0258 (4)	
C11	0.48225 (6)	0.1701 (2)	0.08780 (18)	0.0310 (3)	
H11	0.4701	0.2875	0.1468	0.037*	

### Atomic displacement parameters (Å^2^)

**Table:**

	*U*^11^	*U*^22^	*U*^33^	*U*^12^	*U*^13^	*U*^23^
O1	0.0241 (7)	0.0579 (10)	0.0452 (8)	0.000	0.0121 (6)	0.000
O2	0.0150 (6)	0.0586 (9)	0.0174 (6)	0.000	0.0014 (4)	0.000
C1	0.0199 (9)	0.0522 (14)	0.0409 (11)	0.000	−0.0038 (8)	0.000
C2	0.0220 (9)	0.0336 (11)	0.0365 (10)	0.000	0.0055 (7)	0.000
C3	0.0210 (9)	0.0340 (11)	0.0251 (9)	0.000	0.0029 (7)	0.000
C4	0.0280 (9)	0.0587 (14)	0.0239 (9)	0.000	0.0088 (7)	0.000
C5	0.0314 (10)	0.0656 (15)	0.0178 (8)	0.000	0.0029 (7)	0.000
C6	0.0210 (8)	0.0459 (12)	0.0211 (8)	0.000	−0.0008 (7)	0.000
C7	0.0220 (8)	0.0357 (11)	0.0189 (8)	0.000	0.0036 (6)	0.000
C8	0.0218 (8)	0.0355 (11)	0.0199 (8)	0.000	−0.0004 (6)	0.000
C9	0.0154 (8)	0.0466 (12)	0.0206 (8)	0.000	−0.0001 (6)	0.000
C10	0.0145 (8)	0.0429 (12)	0.0200 (8)	0.000	−0.0003 (6)	0.000
C11	0.0258 (6)	0.0401 (8)	0.0274 (7)	0.0016 (6)	0.0044 (5)	−0.0037 (6)

### Geometric parameters (Å, °)

**Table:**

O1—C2	1.218 (2)	C5—C6	1.393 (2)
O2—C7	1.3685 (19)	C5—H5	0.9500
O2—C9	1.4382 (19)	C6—C7	1.388 (2)
C1—C2	1.506 (3)	C6—H6	0.9500
C1—H1A	0.9800	C7—C8	1.394 (2)
C1—H1B	0.9800	C8—H8	0.9500
C1—H1C	0.9800	C9—C10	1.503 (2)
C2—C3	1.493 (2)	C9—H9A	0.9900
C3—C8	1.383 (2)	C10—C11	1.3844 (16)
C3—C4	1.402 (3)	C10—C11^i^	1.3844 (16)
C4—C5	1.375 (3)	C11—C11^ii^	1.397 (2)
C4—H4	0.9500	C11—H11	0.9500
			
C7—O2—C9	116.56 (12)	C7—C6—C5	119.27 (16)
C2—C1—H1A	109.5	C7—C6—H6	120.4
C2—C1—H1B	109.5	C5—C6—H6	120.4
H1A—C1—H1B	109.5	O2—C7—C6	124.68 (15)
C2—C1—H1C	109.5	O2—C7—C8	115.34 (14)
H1A—C1—H1C	109.5	C6—C7—C8	119.98 (15)
H1B—C1—H1C	109.5	C3—C8—C7	120.52 (15)
O1—C2—C3	120.47 (17)	C3—C8—H8	119.7
O1—C2—C1	120.54 (17)	C7—C8—H8	119.7
C3—C2—C1	118.99 (16)	O2—C9—C10	108.37 (13)
C8—C3—C4	119.34 (16)	O2—C9—H9A	110.0
C8—C3—C2	121.66 (16)	C10—C9—H9A	110.0
C4—C3—C2	119.00 (16)	C11—C10—C11^i^	119.26 (16)
C5—C4—C3	119.93 (17)	C11—C10—C9	120.36 (8)
C5—C4—H4	120.0	C11^i^—C10—C9	120.36 (8)
C3—C4—H4	120.0	C10—C11—C11^ii^	120.37 (8)
C4—C5—C6	120.95 (17)	C10—C11—H11	119.8
C4—C5—H5	119.5	C11^ii^—C11—H11	119.8
C6—C5—H5	119.5		

Symmetry codes: (i) *x*, −*y*, *z*; (ii) −*x*+1, *y*, −*z*.

### Hydrogen-bond geometry (Å, °)

**Table:**

*Cg* is the centroid of the central benzene ring.

**Table:**

*D*—H···*A*	*D*—H	H···*A*	*D*···*A*	*D*—H···*A*
C5—H5···O2^iii^	0.95	2.50	3.435 (2)	170
C11—H11···O1^iv^	0.95	2.54	3.4215 (16)	155
C6—H6···Cg^iii^	0.95	2.95	3.8469 (19)	157

Symmetry codes: (iii) *x*, *y*, *z*+1; (iv) −*x*+1/2, *y*+1/2, −*z*+1.
